# The Cambridge Intensive Weight Management Programme Appears to Promote Weight Loss and Reduce the Need for Bariatric Surgery in Obese Adults

**DOI:** 10.3389/fnut.2018.00054

**Published:** 2018-07-12

**Authors:** Rajna Golubic, Celia Laur, Megan Kelsey, Alana Livesy, Joanna Hoensch, Adrian Park, Sumantra Ray

**Affiliations:** ^1^Addenbrooke's Hospital, Cambridge University Hospitals NHS Foundation Trust, Cambridge, United Kingdom; ^2^NNEdPro Global Centre for Nutrition and Health, St John's Innovation Centre, Cambridge, United Kingdom; ^3^Faculty of Applied Health Sciences, University of Waterloo, Waterloo, ON, Canada

**Keywords:** obesity, diabetes mellitus, hypertension, policy making, bariatric surgery, behavior change, dietary intervention, physical activity adherence

## Abstract

**Objectives:** To investigate the impact of the Cambridge Intensive Weight Management Programme (IWMP) on weight change, eligibility for bariatric surgery, HbA1c, and blood pressure.

**Design:** Prospective non-randomized intervention.

**Setting:** The IWMP is a multi-disciplinary weight loss intervention for severely obese patients to avoid or optimize their physiological state thus enabling bariatric surgery. It uses dietary interventions, pharmacotherapy, and physical activity along with behavior change counseling.

**Participants:** Severely obese patients (Body Mass Index, BMI≥40 kg/m^2^).

**Interventions:** IWMP is a prospective intervention conducted in a National Health Service Tier 3 obesity service. It includes 3 phases of 8 weeks each: weight loss, weight stabilization, and weight maintenance. In each phase, patients adhered to a prescribed dietary regime and attended regular clinic visits. Data included in this analysis are from those who enrolled in IWMP between 2009 and 2013.

**Primary and secondary measures:** The primary outcome was weight change between baseline and completion of the programme. Secondary outcomes included changes in blood pressure, HbA1c and eligibility for bariatric surgery pre-assessment. Changes in outcomes were compared by age, sex, smoking status, and employment.

**Results:** Of *n* = 222 eligible patients, complete data were available for *n* = 141 patients (63.5%). At baseline, the mean (SD) BMI was 49.7 (9.2) kg/m^2^ for women, and 47.9 (7.2) kg/m^2^ for men. Mean (SD) weight change for women was −18.64 (8.36) kg and −22.46 (10.98) kg for men. *N* = 97 (69%) of patients achieved ≥10% weight loss. Individuals aged ≤ 50 years lost significantly more weight than those aged >50 years [mean (SD) weight loss: 22.18 (10.9) kg vs. 18.32 (7.92) kg, *p* = 0.020]. Changes in weight were non-significant by smoking status or employment. Median (IQR) change in systolic and diastolic blood pressure was −6 (−14.6) mmHg and 0 (−8.6) mmHg (non-significant), respectively. There was ~50% reduction in the need for bariatric surgery.

**Conclusions:** For the majority of the patients, IWMP is promoting weight loss and allowing for avoidance of, or optimization before, bariatric surgery.

## Introduction

Obesity and related disorders, such as type 2 diabetes, are reaching epidemic prevalence worldwide (1). In England, the prevalence of obesity increased from 15% in 1993 to 26% in 2014 (2). For severe obesity (defined as body mass index (BMI) ≥40 kg/m^2^ according to the World Health Organization), the prevalence has more than tripled since 1993, with 2% of men and 4% of women being severely obese in 2014 (2). Findings of the Prospective Studies Collaboration suggest that every 5 kg/m^2^ increase in BMI is associated with a 30% higher overall mortality, 40% higher for vascular mortality and 60–120% higher for diabetic, renal and hepatic mortality (3). Evidence also suggests that those who are overweight or obese are at increased risk of developing type 2 diabetes, cardiovascular disease, musculoskeletal disorders and certain cancers (4). Furthermore, people who are obese are seven times more likely to develop diabetes than those within the normal BMI range (5).

Recent literature has demonstrated effectiveness of bariatric (weight loss) surgery for the treatment of obesity. According to a Cochrane review of 22 randomized controlled trials (RCTs) of surgery for weight loss in adults, the direction of effect across studies suggests that those who had surgery had greater weight loss than those who underwent non-surgical management of obesity, one to two years later (6). Improved health-related quality of life and improved diabetes were also reported (6). Picot *et al*. in 2016 suggested that bariatric surgery appears to be a clinically and cost-effective intervention for moderately to severely obese people (4).

Despite the clear NICE guidance (BMI ≥40 kg/m^2^, or between 35 and 40 kg/m^2^ and other significant co-morbidities such as diabetes, hypertension or severe obstructive sleep apnoea that could be improved if they lost weight) (7), the effectiveness of bariatric surgery, and the rising prevalence of obesity, the availability of bariatric surgery in the UK remains poor. Welbourn *et al*. indicated that in the UK, < 1% of individuals who could benefit from bariatric surgery get the treatment (8). One of the potential explanations for this observation is that general practitioners are not able to refer patients directly to surgical services, but instead, there is a tiered system (Tier 1 covers universal services such as health promotion or primary care; Tier 2 covers lifestyle interventions; Tier 3 covers specialist weight management services; and Tier 4 covers bariatric surgery). In the UK, there were 6,032 Finished Consultant Episodes in National Health Services (NHS) hospitals in 2014/15 with a primary diagnosis of obesity and a main or secondary procedure of bariatric surgery (2). Of bariatric surgery patients, 60% were between the ages of 35–54, and 76% of patients were female (2).

In those who are severely obese, intense action may be taken, with support of healthcare professionals, to decrease weight as a way to avoid bariatric surgery or prepare individuals so they are more suitable for surgery (9). The Cambridge Intensive Weight Management Programme (IWMP) is a multi-disciplinary weight loss intervention designed for severely obese patients to lose weight with the support of a multi-disciplinarily healthcare team within a Tier 3 Obesity service. To support this weight loss, the IWMP uses a structured and well-supervised combination of dietary interventions (low calorie diet), pharmacotherapy, and physical activity (written and verbal advice) all underpinned by continual counseling for behavioral change (targeting diet and activity) during 24-week follow-up divided in 3 8-week phases with a different diet composition in each phase.

The aim of the IWMP is to promote weight loss or weight loss maintenance, and behavior changes for people who are severely obese. The ultimate goal for those in IWMP is to avoid bariatric surgery, or to be in a more optimal physiological state prior to bariatric surgery. This paper aims to explain the IWMP and its impact on weight change in severely obese individuals. Secondary measures include: change in eligibility for bariatric surgery, diabetes risk (HbA1c), and blood pressure measured across the strata of age, sex, and employment.

## Methods

### Study population

The IWMP is a prospective multi-component intervention conducted by the Tier 3 Obesity Services, at Addenbrooke's Hospital, Cambridge University Hospitals NHS Foundation Trust, in Cambridge, UK. Although this service is ongoing, data for this intervention was collected from 2009 to 2013.

Patients were recruited from the Addenbrooke's hospital obesity hospital service, which provides healthcare to the patients from East Anglia region. Individuals who were eligible for the IWMP were being treated in the obesity clinic and then agreed to complete the intensive programme with fortnightly clinic visits. The eligibility typically meant that patients were severely obese (BMI ≥40 kg/m^2^) but wanted to avoid bariatric surgery or had been recommended for patient optimization prior to bariatric surgery. Any contraindication to a low energy diet was used as exclusion criteria, including: pregnancy, significant renal or hepatic disease, unstable coronary heart disease, uncontrolled diabetes, active eating disorder, unstable psychological status, or inability to attend clinic visits.

Prior to entering the study, patients underwent a rigorous pre-programme screening that consisted of comprehensive medical and psychological assessments. A detailed medical assessment designed to review any medical or surgical contraindications was required to select eligible patients as well as identify potential obesity-related co-morbidities to be optimized (10). The Epworth Sleepiness Scale was used to assess for the symptoms of obstructive sleep apnoea (11). Psychological assessments were taken prior to the intervention, and throughout the programme, however results will not be presented here. Since this work is based on hospital service data, a specific ethics committee approval was not required.

### Intervention

**Figure 1:**
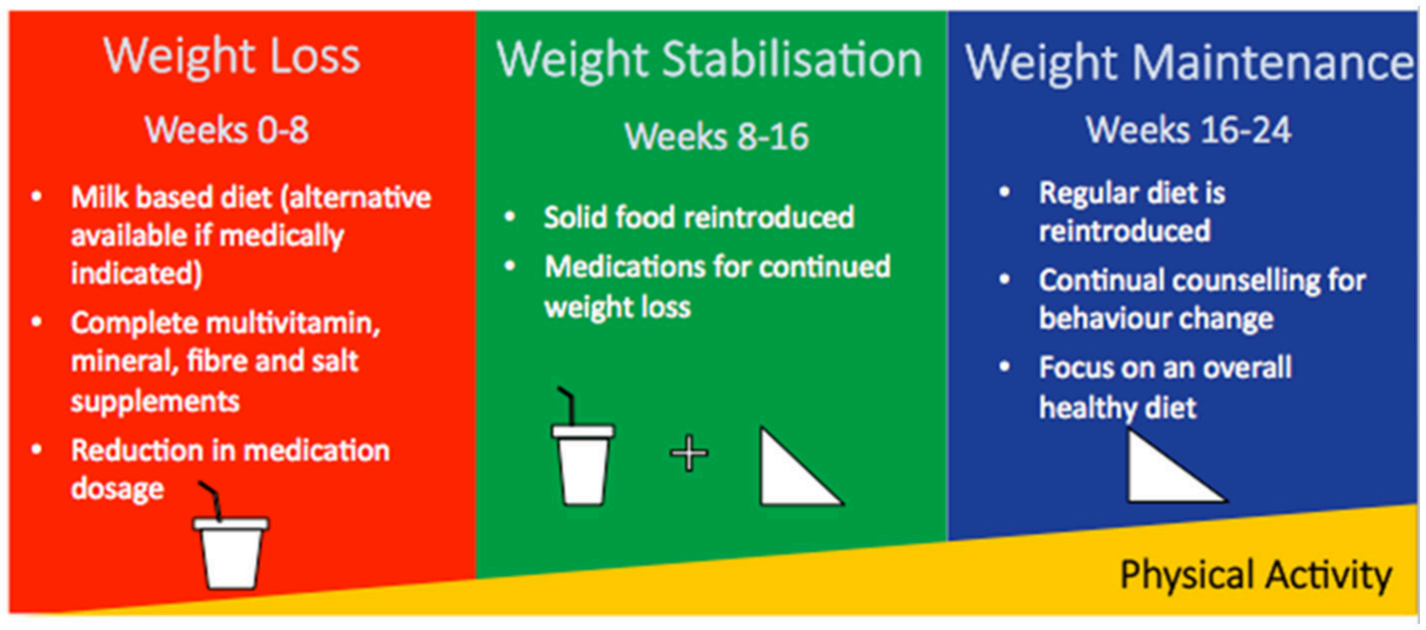
Overview of the 3 phases within the 24-week intensive weight management programme.

The IWMP was delivered by a multi-disciplinary team, which included consultant physicians, a clinical (obesity) psychologist, clinical nurse specialists, advanced specialist dietitians, and specialist dietitians. The IWMP consisted of a 24-week weight loss programme divided into three 8-week phases (Figure 1). In each phase, patients were required to adhere to a prescribed dietary regimen, record food intake and physical activity, and attend regular clinic visits. Additional medical and psychological management of related comorbidities using a multidisciplinary team approach ensure clinical safety for patients enrolled on the programme.

#### Phase 1 (weight loss)

Patients consume an all-liquid diet of 4–5 pints (1,136–1,420 kcal) of semi-skimmed milk per day or 800–1,000 mL of an alternate supplement (1,280–1,600 kcal) of Nutricia Fortisip Extra® if volume is not tolerated. The Nutricia Fortisip Extra® is a dietary supplement used in individuals with malnutrition. The dietary composition of 100 mL of semi- skimmed milk included proteins (3.6 g), carbohydrates (4.8 g, of which sugars 4.8 g), and fat (1.8 g) while 100 mL of Nutricia Fortisip Extra® included proteins (6 g), carbohydrates (18.4 g, of which sugars 6.7 g), and fat (5.8 g). The volume prescribed was dependent on protein requirement (12), ensuring a minimum of 50 g a day to meet with Low Calorie Diet guidelines (13). Patients also took a complete multivitamin and mineral supplement as well as fiber and sodium supplements (in a form of sodium chloride) as the liquid diet is typically low in sodium chloride. Medications for diabetes that may cause hypoglycaemia and antihypertensive medications were reduced in order to minimize risks of hypoglycaemia and symptomatic postural hypotension which might otherwise occur with the lifestyle intervention. There were solid and lactose free choices for those who were unable to tolerate the milk or supplements of the programme.

#### Phase 2 (weight stabilization)

Patients transitioned to a diet of 50% milk and 50% solid food. Weight loss medication was added (Orlistat and Sibutramin).

#### Phase 3 (weight maintenance)

Patients returned to a 100% solid diet and individualized energy-balanced nutritionally complete diet. Patients received guidance on weight maintenance including relapse management and a focus on an overall healthy diet.

#### Follow-up

Patients were followed up for 3 months after completion of the IWMP before care was transferred back to the general practitioners.

### Outcome measures

To monitor patient's progress, the following outcomes were measured at the fortnightly clinic visits: weight (kg), height (m), and blood pressure (mmHg). Weight was measured using standardized Seca® (Hamburg, Germany) weight scales. Height was measured using a Seca® (Hamburg, Germany) stadiometer. BMI was calculated as weight divided by high squared and expressed as kg/m^2^. HbA1c, an indicator of diabetic control, was assessed at the beginning and at the end of the intervention using a standard microprocessor controlled HPLC system dedicated to A1C analysis (TOSOH A1c Analyser). Physical activity was assessed using the Baecke's Questionnaire (14) in a small subset of patients at baseline (results not shown due to small sample size of the participants responding to the questionnaire which is a voluntary part of the multifarious monitoring activities which patients need to conduct in this service). Smoking status was self-reported as a dichotomous variable (smoker/non-smoker). Employment status was self-reported (employed/unemployed/retired) with employed patients also providing details about their occupation (no standard occupation coding was used). The employed patients were further classified according to occupational physical activity (sedentary/non-sedentary job). This classification was performed in an arbitrary fashion based on the common qualitative descriptions of the jobs reported.

### Statistical analysis

As this is a service evaluation of exploratory nature rather than a primary research study, power calculations were not performed nor were strict hypotheses formulated prior to the onset of the IWMP. IWMP is a complex clinical service intervention with tailoring to the needs of individual patients, rather than a controlled study intervention.

Descriptive statistics for continuous variables including weight, height, BMI and HbA1C, are shown as mean (SD) when distribution was normal or median (IQR) in the case of substantial departure from normality, and *N* (%) for categorical variables including smoking, employment, and occupational physical activity. An *a priori* decision was made to present baseline characteristics, and rate in the weight or BMI change, by sex for comparability with the studies that include women or men only. To test the differences in weight change and BMI change across 2 or ≥3 categories of socio-demographic variables, *t*-test and ANOVA were performed, respectively. The Mann-Whitney *U*-test and the Kruskall-Wallis test were conducted to assess the difference in non-normally distributed variables according to 2 or ≥3 categories of socio-demographic variables, respectively. The rate of weight or BMI change between the 3 phases of the programme was assessed using Kruskall-Wallis test.

The above-mentioned socio-demographic variables were considered to be potential predictors of the outcomes of interest. Each predictor was entered in a univariable linear regression model with weight change and BMI change being continuous outcomes in separate models. A *p*-value < 0.10 was considered statistically significant in these univariable models and *p* < 0.05 in all other analyses. Only age-category and sex reached statistical significance and were used in the further multivariable model.

## Results

### Participant characteristics

Baseline characteristics are shown in Table 1. Of *n* = 222 eligible patients, complete data were available for *n* = 141 patients (63.5%), with 48% women (Table 1). The average age was 47 years and 50.5 years of age for women and men, respectively. All participants qualified as severely obese with the mean (SD) BMI at 49.7 (9.2) and 47.9 (7.2) kg/m^2^ for women and men, respectively. Sixty-six percent of men and 73% of women had a confirmed diagnosis of type 2 diabetes. Follow-up data was collected, however responses were only received from *N* = 45 at 3 months and *n* = 0 at 6 months. The prevalence of unemployment was high, with 25% (*N* = 12) in men and 42% (*N* = 22) in women. *N* = 8 (17%) men and *N* = 10 (19%) women reported being current smokers.

**Table 1:** Baseline characteristics by sex.

	**Men (*N* = 74)**	**Women (*N* = 67)**
	**Mean (*SD*)**	**Mean (*SD*)**
Age (y)	47.0 (10.7)	50.5 (12.1)
Weight (kg)	151.7 (26.9)	132.8 (29.2)
BMI (kg/m^2^)	47.9 (7.2)	49.7 (9.2)
Systolic blood pressure (mmHg)	129.5 (16.9)	122.9 (16.9)
Diastolic blood pressure (mmHg)	72.5 (12.1)	71.6 (12.1)
HbA1c (%)	8.63 (1.75)	7.7 (1.6)
Type 2 diabetes diagnosis, *N* (%)	44 (66%)	54 (73%)
**EMPLOYMENT STATUS**		
Employed, *N* (%)	31 (63%)	26 (50%)
Unemployed, *N* (%)	12 (25%)	22 (42%)
Retired, *N* (%)	6 (12%)	4 (8%)
**SMOKING STATUS**		
Non-smoker, *N* (%)	40 (83%)	42 (81%)
Smoker, *N* (%)	8 (17%)	10 (19%)

*Data on employment and smoking status were available for 101 and 100 patients, respectively*.

### Primary outcome—weight change

There was an average (SD) weight loss of 20.5 (9.8) kg following completion of the intervention. Sixty-nine percent (*N* = 97) had lost 10% or more of their body weight.

Comparisons between weight loss, percentage weight loss, and BMI change are presented in Table 2, stratified by sex, smoking status and employment status. A statistically significant difference was found in mean (SD) weight loss between men and women with men losing more weight than women [women: 18.64 (8.36) kg; men: 22.46 (10.98) kg, *p* = 0.021]. This did not remain significant for BMI change and percent weight loss. Significance was also found for age group, with results suggesting that patients who are 50 years old or less have greater mean weight loss [ ≤ 50 years, 22.18 (10.9) kg; >50 years, 18.32 (7.92) kg, *p* = 0.020] and mean BMI change [ ≤ 50 years, −7.58 (3.45) kg/m^2^; >50 years, −6.26 (2.78) kg/m^2^, *p* = 0.016] than their older counterparts. There were no significant differences in weight loss, percentage weight loss, or BMI change according to smoking or employment status.

**Table 2:** Mean weight loss, percentage weight loss, and BMI change by sex, age, smoking status, and employment status.

	**Weight loss (kg)**	**% Weight loss**	**BMI change**
	**Mean *(SD)***	**Mean *(SD)***	**Mean *(SD)***
**SEX**			
Women (*n* = 74)	18.64 (8.36)	14.08 (6.19)	−6.94 (3.19)
Men (*n* = 67)	22.46 (10.98)	14.59 (6.55)	−7.04 (3.28)
*p*-value	0.021	0.634	0.849
**AGE GROUP**			
≤50 years (*n* = 77)	22.18 (10.9)	15.22 (6.97)	−7.58 (3.45)
>50 years (*n* = 62)	18.32 (7.92)	13.21 (5.31)	−6.26 (2.78)
*p*-value	0.020	0.062	0.016
**SMOKING STATUS**			
Non-smoker (*n* = 81)	22.20 (10.05)	15.1 (6.57)	−7.51 (3.35)
Smoker (*n* = 18)	19.54 (13.12)	13.3 (7.08)	−6.71 (7.08)
*p*-value	0.339	0.263	0.377
**EMPLOYMENT STATUS**			
Employed (*n* = 57)	22.52 (12.08)	14.7 (7.38)	−7.45 (3.74)
Unemployed (*n* = 34)	20.42 (8.73)	14.4 (6.38)	−7.31 (3.20)
Retired (*n* = 9)	19.09 (4.43)	14.9 (3.77)	−6.27 (1.98)
*p*-value	0.495	0.967	0.635

The rate of weight change is presented in Table 3, including the three phases of IWMP. There was a significant difference in the rate of weight change between the phases (*p* < 0.001). The highest rate of weight change was observed in the first phase of the intervention with median (IQR) −11.2 (−14.7, −8.2) kg. The magnitude of the change decreased to −4.8 (−7.1, 2.4) kg and −1.2 (−3.2, 0.2) kg in the second and third phase, respectively.

**Table 3:** Rate of weight change (kg) throughout the phases of the programme.

**Phase**	**Mean**	***SD***	**Median**	**Interquartile range 25th; 75th percentiles**
Weeks 1–8	−11.9	7.5	−11.2	−14.7; −8.2
Weeks 9–16	−4.8	4.3	−4.8	−7.1; −2.4
Weeks 17–24	0.1	12.7	−1.2	−3.2; 0.2

*p < 0.001 for the Kruskal-Wallis test across the 3 phases*.

**Figure 2:**
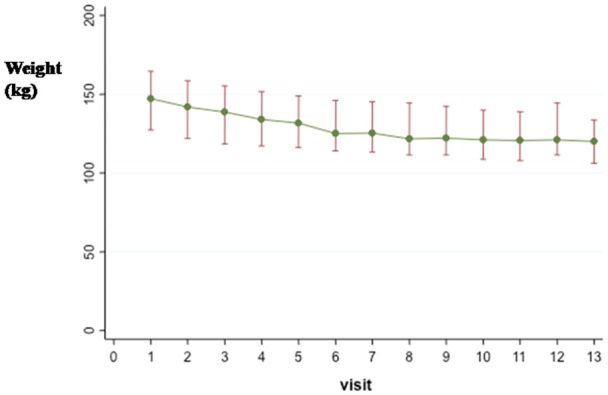
Median (IQR) body weight (kg) by visit and at the 3-month follow-up (visit 13).

Figure 2 details the median (IQR) weight for every visit and 3-month follow-up (visit 13 in the figure). The median (IQR) body weight dropped from 147.2 (127.4, 164.6) kg at baseline to 121.0 (111.4, 144.4) kg at the end of the third phase and reached 120.0 (106.2, 133.6) kg at the 3-month follow-up. The greatest weight reduction was noted in the first month of the study and was followed by a slower weight loss and finally stabilization. Similar trends were observed for BMI (Supplementary Information, Figure 1).

Univariate regression models with weight loss as the outcome showed significance for sex and age group (Table 4). These two predictors were then entered in a multivariable (i.e., mutually adjusted) model and the significance remained for both predictors with the same direction and similar magnitude of the association as in univariate models, i.e., male sex (β = 4.32, 95% CI: 1.14–7.50) and being younger than 50y (β = 4.36, 95% CI: 1.16–7.55) were associated with significantly greater weight loss compared to being female of older than 50y (Supplementary Information Table 1). This model suggests independent associations of age and sex with weight loss. As no statistical significance was reached in univariate models with other socio-demographic variables, these were not entered in the multivariable model and their effects on the weight loss are interpreted only from univariate models.

**Table 4:** Univariable linear regression models assessing the associations of sex, age, smoking status, and occupation with weight loss (IWMP, baseline *N* = 141).

	**β-coefficient**	**95% Confidence interval**	***p*-value**
Sex (male)	3.82	0.59 – 7.05	0.021
Age (≤ 50y)	3.85	0.61 – 7.10	0.020
Smoking (yes)	−2.67	−8.17 – 2.84	0.339
Occupation (Employed)	2.10	−2.43 – 6.63	0.359
Occupation (Retired)	−1.331	−8.85 – 6.19	0.726

*NB, the following reference categories were used: sex- female, age–>50 years, smoking- no, occupation- unemployed*.

### Secondary outcomes

#### Bariatric surgery

At the time of analysis, bariatric surgery guidelines for the East of England Specialised Commissioning Group indicated that eligible patients must have a BMI ≥40 kg/m^2^ with either type 2 diabetes or severe obstructive sleep apnoea. Of the *n* = 32 IWMP patients who met the BMI criteria for bariatric surgery at baseline, only *n* = 17 were still eligible by the end of the intervention (46.7% reduction). Of the *n* = 26 patients who met the BMI and age criteria (18–65 years) for bariatric surgery (15), only *n* = 13 were still eligible at post-intervention (50% reduction).

#### Diabetes (HbA1c)

Of the patients with diabetes (*n* = 66 for type 2 diabetes; *n* = 2 for type 1 diabetes) there was a median (IQR) reduction in HbA1c of 8.6 (0–17.8)%. This equates to a median (IQR) unit reduction of HbA1c of 0.6% (0–1.6) according to Diabetes Control and Complications Trial (DCCT) and −8.0 (−17.0, −1.0) mmol/mol according to the International Federation for Clinical Chemistry (IFCC). A greater reduction in HbA1C was observed among men, those older than 50 years, non-smokers and retired compared to the counterparts without these characteristics (Supplementary Information, Table 2). However, none of these differences were statistically significant (Supplementary Information, Table 2).

#### Blood pressure

There were no significant changes in blood pressure outcomes based on demographic variables assessed. The median (IQR) change in systolic and diastolic blood pressure was−6 (−14 to 6) and 0 (−8 to 6) mmHg, respectively (Supplementary Information, Table 3, Supplementary Information, Figures 2, 3).

## Discussion

The findings of this intervention in severely obese individuals suggest that the completion of the IWMP is associated with an average (SD) weight loss of 20.5 (9.8) kg over 24 weeks. Sixty-nine percent (*n* = 97) of the patients had lost 10% or more of their body weight. Sex and age were significant independent predictors of weight loss, with men and those younger than age 50 achieving significantly greater weight loss than their counterparts. The significant weight loss associated with the IWMP shows the potential of this programme to help severely obese patients. It must remain clear that this programme must only be followed under strict supervision of several healthcare providers working as a multidisciplinary team, and it is not advised for individuals to follow this regime on their own. As the number of severely obese people increases, more programmes must be available to help them lose weight to help avoid bariatric surgery or to optimize patients before surgery, thus reducing deleterious health effects of obesity.

### Clinical significance

The clinical effectiveness of the IWMP is demonstrated by the substantial weight loss, which is greater than in some similar weight management programmes, as described below. Although not statistically significant, there was also a clinically appreciable reduction in HbA1c at individual patient level. There was a substantial drop in the need for bariatric surgery in relation to the local referral guidelines. Although the absolute magnitude of the reduction in systolic blood pressure was small and did not reach statistical significance, there was an overall decrease in the use of antihypertensive medications.

Many people in IWMP are on several medications, however due to the nature of the data, there was no way to quantify medication change in a consistent way. Experiences of IWMP clinicians indicate an overall reduction in hypertension medication. Since blood pressure remained unaffected despite this reduction in medication, the lack of change may indicate a positive outcome. Although not discussed due to the small sample size, observation from IWMP clinicians indicate the IWMP has been effective in controlling obstructive sleep apnoea and to an extent hypercholesterolemia.

### Comparison with other weight management programmes

The Danish RCT, the CAROT study (16) (*N* = 192) found that low energy diet (LED; 800–1,200 kcal/day) and very low energy diet (VLED; < 800 kcal/day) resulted in an average weight loss of 10.7 kg and 11.4 kg over 16 weeks, respectively which is a smaller weight reduction than IWMP. The observed differences could be explained by the differences in the baseline characteristics of the patients (those in the CAROT were somewhat older [mean age 63 years] and leaner [mean (SD) for baseline weight was 103.2 (15.0) kg and for baseline BMI was 37.3 (4.8) kg/m^2^] compared to the IWMP) (16). Furthermore, the different nature (i.e., dietary content) and the duration of the interventions (16 weeks for the CAROT vs. 24 weeks for the IWMP) could possibly account for the observed differences in the weight loss (16).

A feasibility study (*N* = 91) in the primary care setting in the UK (17) with similar baseline characteristics as the IWMP reported a mean (SD) weight loss of 14.7 (10.8) kg over the period of 12 months. The study used the same 3 phases as the IWMP (weight loss with low energy liquid diet for 12 weeks, food reintroduction for 6–8 weeks and weight maintenance until 12 months) but the intervention lasted for one year.

Recently published results of the DiRECT trial (18) have demonstrated that the primary care intervention comprising of total diet replacement (825–853 kcal/day formula diet for 3–5 months), stepped food reintroduction (2–8 weeks), withdrawal of antidiabetic and antihypertensive medications and structured support for long-term weight loss maintenance was associated with a remission to a non-diabetic state after 12 months among obese adults with type 2 diabetes. This study indicates that effective weight management interventions may be performed in primary care thus translating the findings from the tertiary centers to the community.

In a review of very low calorie or low-energy liquid-formula diets in people with and without type 2 diabetes, all trials reported weight loss for all participants, with our without diabetes (19). In another review focused on people with diabetes, the mean weight loss from the 17 studies was 13.2 kg (range 4.1–24 kg), with the duration ranging from 5 days to 6 months (20). In the STAMPEDE trial, Schauer et al, demonstrated that in obese patients with type 2 diabetes, bariatric surgery plus intensive medical therapy was more effective than medical therapy alone (21).

### Strengths and limitations

The strengths of this study include the unique multidisciplinary team approach with fortnightly follow-ups and support to the patients with severe obesity. As this is a service-based intervention, it is likely that the majority of the individuals with severe obesity in the geographic region which the hospital serves have been captured, thus increasing generalisability of our findings. As this is a service based intervention it was not possible to have a control group, thus there is no randomization of participants.

As this programme is conducted in a clinical NHS setting with a multi-faceted team over several years, there are changing bariatric surgery criteria [lower BMI cut-offs for the consideration of bariatric surgery, i.e., 35 kg/m^2^ in those with diabetes or significant co-morbidities including hypertension, or obstructive sleep apnoea 15], as well as changes to staffing and procedures. These are all limiting factors of data collection. Some of the available data was not sufficient for analysis due to missing data or inconsistent entry, thus limiting sample size. Further analysis may be conducted to examine the changes in medications and psychological status, however this is currently outside the remit of this study.

As with any participant intensive study, it is challenging to determine how well participants followed the diet and physical activity recommendations. The pre-determined commitment, the fortnightly clinic visits, and diet and physical activity records were all strategies used to measure adherence, however it is still difficult to account for this factor. There is also no objective measure of physical activity.

Although follow-up with participants was attempted at 3 months, there was a minimal response rate and thus follow-up data is lacking. Beyond 3 months, there was no follow-up. Due to this lack of follow-up, it was not possible to assess the potential weight regain after post-intervention weight loss. Reviewing long term weight loss maintenance is an important next step for IWMP.

It is widely acknowledged that screening for various psychological measures (including depression and anxiety, self-esteem, eating behavior and attitudes, and quality of life) should be used as part of weight management assessment. IWMP used standardized and validated instruments (11, 22–25) to measure each of these constructs. However, we did not include them in this analysis due to missing data for a large proportion of the participants.

As this is a small group of patients, it is difficult to have studies with a large sample size. Data were collected to monitor individual progress, and when viewed together the sample size may appear small. For this reason, analysis focused on weight change and a small number of secondary outcomes. Furthermore, we identified only age and sex to be significant predictors of weight loss thus limiting the multivariable analysis for other factors considered to be possibly associated with weight loss.

## Conclusion

The IWMP is a service designed for severely obese individuals who have the ultimate goal of avoiding the need for bariatric surgery or to optimize the patient before surgery. IWMP patients lost an average (SD) of 20.5 (9.8) kg over the course of an intensive 24-week programme. There was around a 50% reduction in the need for bariatric surgery. When used appropriately under direct professional supervision, the IWMP has the potential to significantly decrease weight of severely obese individuals. Further research into the clinical effectiveness of IWMP would include the addition of long term follow-up to assess weight maintenance as well as measures of vascular function and genotype to assess inter-individual difference in physiological and clinical response to the IWMP intervention.

## Author contributions

RG analyzed data, drafted the manuscript and made subsequent revisions according to co-authors' comments. CL contributed to data collection and manuscript preparation. MK performed part of the analysis. JH lead data collection and study management. AP conceived the study, oversaw all stages of the project, provided critical input to all drafts of the manuscript. SR conceived the study, oversaw all stages of the project, provided critical input to all drafts of the manuscript. AL contributed to data collection.

### Conflict of interest statement

The authors declare that the research was conducted in the absence of any commercial or financial relationships that could be construed as a potential conflict of interest.
