# Supplementary material for: The Cambridge Intensive Weight Management Programme Appears to Promote Weight Loss and Reduce the Need for Bariatric Surgery in Obese Adults
Source: Front Nutr. 2018 Jul 12;5:54. doi: 10.3389/fnut.2018.00054 (PMC6052095; doi:10.3389/fnut.2018.00054)
Supplement: Supplementary file 1 [file Table_1.DOC]

**Supplementary Information, Table 1:** Age- and sex-adjusted linear regression model with weight loss as outcome (IWMP, baseline N=141)

| **Weight Loss** | **β-coefficient** | **95% Confidence Interval** | **p-value** |
| --- | --- | --- | --- |
| Sex (male) | 4.32 | 1.14 – 7.50 | 0.008 |
| Age (≤50) | 4.36 | 1.16– 7.55 | 0.008 |
