# Supplementary material for: The Cambridge Intensive Weight Management Programme Appears to Promote Weight Loss and Reduce the Need for Bariatric Surgery in Obese Adults
Source: Front Nutr. 2018 Jul 12;5:54. doi: 10.3389/fnut.2018.00054 (PMC6052095; doi:10.3389/fnut.2018.00054)
Supplement: Supplementary file 2 [file Table_2.DOC]

**Supplementary Information, Table 2:Variation in HbA1c according to sociodemographic parameters**

|  | **Change in HbA1c (unit (%) change, DCCT)**  ***Median (IQR)*** | **Change in HbA1c (%)**  ***Median (IQR)*** | **Change in HbA1c (unit (mmol/mol) change, IFCC)**  ***Median (IQR)*** |
| --- | --- | --- | --- |
| **Sex** |  |  |  |
| Women (n=28) | -0.5 (-1.4, -0.1) | -7.5 (-16.8, -1.3) | -6.0 (-15.0, -1.0) |
| Men (n=28) | -0.9 (-1.9, 0.1) | -11.03 (-20.2, 1.3) | -9.0 (-20.5, 1.0) |
| p-value | 0.742 | 0.819 | 0.780 |
| **Age group** |  |  |  |
| ≤50 years (n=39) | -0.4 (-1.6, 0) | -6.8 (-16.6, 0) | -4.5 (-17.0, 0) |
| >50 years (n=21) | -0.9 (-1.6, -0.2) | -13.2 (-19.3, -3.0) | -10.0 (-17.0, -7.0) |
| p-value | 0.502 | 0.394 | 0.460 |
| **Smoking status** |  |  |  |
| Non-smoker (n=7) | -0.7 (-1.3, -0.2) | -9.7 (-17.3, -2.6) | -7.5 (-17.0, -2.0) |
| Smoker (n=34) | -0.2 (-1.8, 1.0) | -3.2 (-16.7, 12.7) | -2.0 (-19.0, 11.0) |
| p-value | 0.267 | 0.200 | 0.267 |
| **Employment Status** |  |  |  |
| Employed (n=25) | -0.5 (-1.3, -0.2) | -7.4 (-17.3, -2.6) | -4.5 (-13.0, 1.0) |
| Unemployed (n=14) | -0.4 (-1.2, 0.1) | -6.0 (-16.9, 1.5) | -6.0 (-14.0, -2.0) |
| Retired (n=5) | -1.4 (-1.5, -0.9) | -13.7 (-16.7, -11.3) | -15.0 (-16.0, -10.0) |
| p-value | 0.734 | 0.962 | 0.731 |

Abbreviations: DCCT- Diabetes Control and Complications Trial, IFCC- International Federation of Clinical Chemistry
