# Supplementary material for: The Cambridge Intensive Weight Management Programme Appears to Promote Weight Loss and Reduce the Need for Bariatric Surgery in Obese Adults
Source: Front Nutr. 2018 Jul 12;5:54. doi: 10.3389/fnut.2018.00054 (PMC6052095; doi:10.3389/fnut.2018.00054)
Supplement: Supplementary file 3 [file Table_3.DOC]

**Supplementary Information, Table 3: Variation in blood pressure according to sociodemographic parameters**

|  | **Change in systolic blood pressure (mmHg)**  ***Median (IQR)*** | **Change in diastolic blood pressure (mmHg)**  ***Median (IQR)*** |
| --- | --- | --- |
| **Sex** |  |  |
| Women (n=23) | 2 (-10, 20) | 0 (-6, 10) |
| Men (n=37) | -10 (-17, 4) | 0 (-10, 6) |
| p-value | 0.100 | 0.393 |
| **Age group** |  |  |
| ≤50 years (n=21) | -7 (-15, 6) | 0 (-10, 6) |
| >50 years (n=39) | -4 (-14, 10) | 0 (-8, 6) |
| p-value | 0.603 | 0.715 |
| **Smoking status** |  |  |
| Non-smoker (n=42) | -4 (-14, 12) | 0 (-10, 8) |
| Smoker (n=2) | -25 (-26, -24) | -6 (-6, -6) |
| p-value | 0.071 | 0.572 |
| **Employment Status** |  |  |
| Employed (n=26) | -10 (-18, 6) | 0 (-10, 8) |
| Unemployed (n=13) | 7 (-7, 24) | 1 (-10, 9) |
| Retired (n=7) | -10 (-10, 4) | -2 (-7, 10) |
| p-value | 0.083 | 0.991 |

Exposure information missing data in this analysis:

N=1 for sex, N=1 for age, N=17 for smoking status and N=16 for employment status
