# Supplementary material for: The Cambridge Intensive Weight Management Programme Appears to Promote Weight Loss and Reduce the Need for Bariatric Surgery in Obese Adults
Source: Front Nutr. 2018 Jul 12;5:54. doi: 10.3389/fnut.2018.00054 (PMC6052095; doi:10.3389/fnut.2018.00054)
Supplement: Supplementary file 4 [file Image_1.pdf]

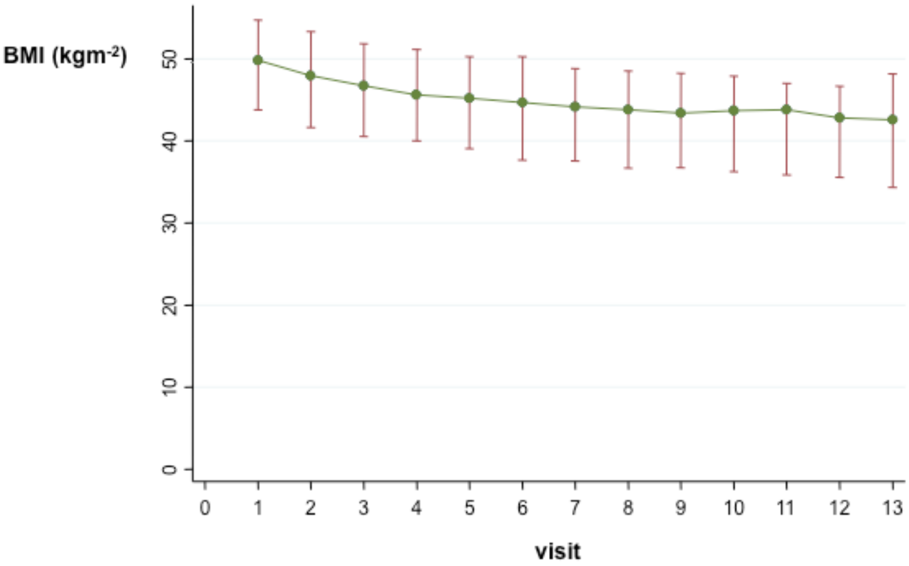

Supplementary Information, Figure 1: Median (IQR) BMI (kg/m^2) by visit and at the 3 month follow-up (visit 13)
