# Supplementary figures and images for: The Cambridge Intensive Weight Management Programme Appears to Promote Weight Loss and Reduce the Need for Bariatric Surgery in Obese Adults
Source: Front Nutr. 2018 Jul 12;5:54. doi: 10.3389/fnut.2018.00054 (PMC6052095; doi:10.3389/fnut.2018.00054)

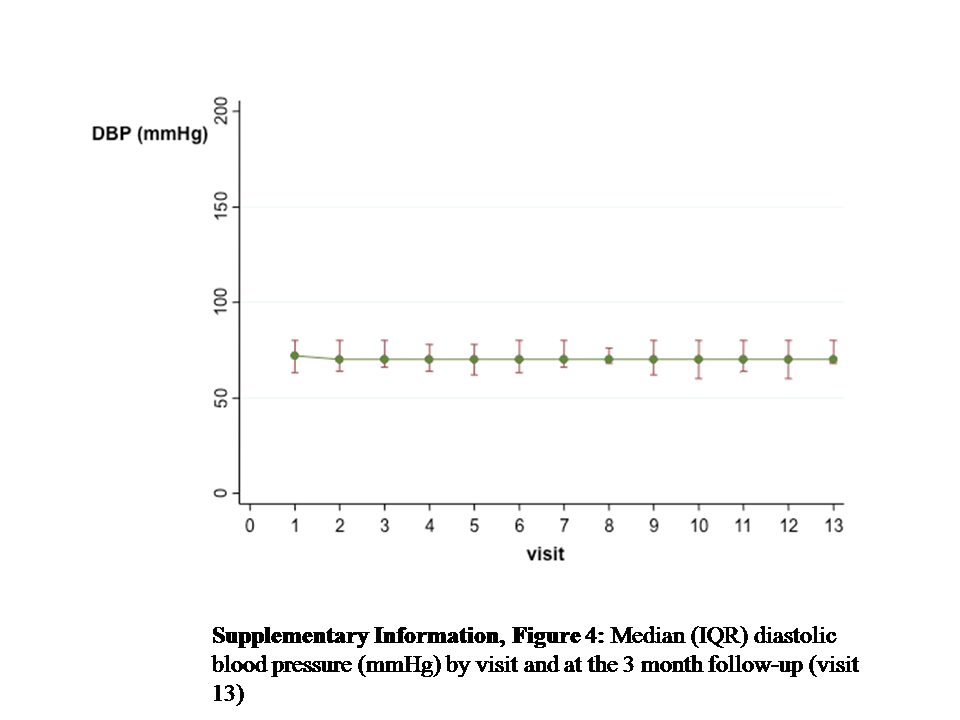

Supplement: Supplementary file 7 [file Image_4.tif]
